# A survey of motif discovery methods in an integrated framework

**DOI:** 10.1186/1745-6150-1-11

**Published:** 2006-04-06

**Authors:** Geir Kjetil Sandve, Finn Drabløs

**Affiliations:** 1 Department of Computer and Information Science, NTNU – Norwegian University of Science and Technology, N-7052, Trondheim, Norway; 2 Department of Cancer Research and Molecular Medicine, NTNU – Norwegian University of Science and Technology, N-7006, Trondheim, Norway

## Abstract

**Background:**

There has been a growing interest in computational discovery of regulatory elements, and a multitude of motif discovery methods have been proposed. Computational motif discovery has been used with some success in simple organisms like yeast. However, as we move to higher organisms with more complex genomes, more sensitive methods are needed. Several recent methods try to integrate additional sources of information, including microarray experiments (gene expression and ChlP-chip). There is also a growing awareness that regulatory elements work in combination, and that this combinatorial behavior must be modeled for successful motif discovery. However, the multitude of methods and approaches makes it difficult to get a good understanding of the current status of the field.

**Results:**

This paper presents a survey of methods for motif discovery in DNA, based on a structured and well defined framework that integrates all relevant elements. Existing methods are discussed according to this framework.

**Conclusion:**

The survey shows that although no single method takes all relevant elements into consideration, a very large number of different models treating the various elements separately have been tried. Very often the choices that have been made are not explicitly stated, making it difficult to compare different implementations. Also, the tests that have been used are often not comparable. Therefore, a stringent framework and improved test methods are needed to evaluate the different approaches in order to conclude which ones are most promising.

**Reviewers: **This article was reviewed by Eugene V. Koonin, Philipp Bucher (nominated by Mikhail Gelfand) and Frank Eisenhaber.

## Open peer review

Reviewed by Eugene V. Koonin, Philipp Bucher (nominated by Mikhail Gelfand) and Frank Eisenhaber. For the full reviews, please go to the Reviewers' comments section.

## Introduction

Understanding the regulatory networks of higher organisms is one of the main challenges of functional genomics. Gene expression is regulated by transcription factors (TF) binding to specific transcription factor binding sites (TFBS) in regulatory regions associated with genes or gene clusters. Identification of regulatory regions and binding sites is a prerequisite for understanding gene regulation, and as experimental identification and verification of such elements is challenging, much effort has been put into the development of computational approaches. Good computational methods can potentially provide high-quality prediction of binding sites and reduce the time needed for experimental verification. However, the computational approach has turned out to be at least as challenging as the experimental one, and a very large number of different methods have been developed.

Computational discovery of regulatory elements is mainly possible because they occur several times in the same genome, and because they may be evolutionary conserved. This means that novel regulatory elements may be discovered by searching for overrepresented motifs across regulatory regions. However, this apparently simple approach is complicated by the fact that most binding site motifs are short, and they may also show some sequence variation without loss of function. Therefore most motifs are also found as random hits throughout the genome, and it is a challenging problem to distinguish between these false positive hits and true binding sites.

One of the early origins of DNA motif discovery is the computer program written in 1977 by Korn *et al*. [1] that was able to discover sequence similarities in regions immediately upstream of TSS. Both mismatches and flexible gaps were accounted for, but using only pairwise comparisons. This approach was further developed by Queen *et al*. [2], comparing multiple sequences simultaneously. In this work, the exact requirements of a motif was also defined clearly, with quorum constraints on sequence support, max number of mismatches in occurrences, and max distances between occurrence positions in the different sequences. In the same year, Stormo *et al*. [3] introduced a Perceptron algorithm that calculated the sum of independent weighted match scores for each position of a motif aligned with a sequence. Similar to this, Staden [4] introduced a position weight matrix with weights corresponding to log-frequencies of nucleotides in aligned motif occurrences. A very nice historical account of the early development of motif models is given in [5].

The most common approach to *de novo *computational discovery of regulatory elements is to extract a set of sequences from the genome, typically fixed size upstream regions for a set of genes having e.g. similar functional annotation or gene expression. An algorithm is then used to discover the most overrepresented motifs according to some motif model and statistical measure.

Several extensions to this basic approach may be used to increase its sensitivity, by including additional prior knowledge about gene regulation. Regulatory elements are not randomly distributed, but tend to form clusters of regulatory modules. The context of putative regulatory elements may also be important, such as other nearby elements, the presence of CpG-islands, or the position in the overall DNA structure. Individual genes in a gene set may show different levels of co-regulation e.g. in a microarray experiment, and this may be used as a weight function to increase the influence from potentially important genes. Finally, additional sources of information, such as regulatory regions of orthologous genes, will often be available.

More than a hundred methods have been proposed for motif discovery in recent years, representing a large variation with respect to both algorithmic approaches as well as the underlying models of regulatory regions. There is also large variation regarding how methods are described and tested, making it even harder to get a good overview of the field. Many reviews of motif discovery methods have therefore been written, with varying focus and intended audience. The recent review by Pavesi *et al*. [6] is a very accessible and broad introduction to the field. It divides methods into consensus- and alignment-based, and surveys the most established methods one at a time. It also discusses background modeling, evaluation of motifs and the practicalities of using these methods. The review by Wasserman and Krivan [7] has a stronger focus on the underlying biology of motif discovery in regulatory regions. It also goes a bit more into the combinatorial nature of binding sites, and touches upon issues such as phylogenetic footprinting, CpG-islands and chromatin structure. Finally, some reviews focus on specific techniques such as phylogenetic footprinting [8], or on specific genomes [9].

Here we present a structured framework for describing motif discovery methods, where we focus on the modeling of regulatory regions, in particular in eukaryote genomes, and with a finer level of detail compared to previous surveys. The emphasis is on how the multiple binding sites for modules of combinatorially acting regulatory elements can be modeled, and how additional data sources may be integrated into such models.

Our framework allows for a systematic and quite exhaustive survey of recent methods. Here we survey methods with respect to individual elements of our model, which makes it easier to spot important differences and similarities between methods. Furthermore, this approach reveals important differences between methods on aspects that in most papers are not discussed as deliberate choices. Relevant examples are how matching scores of several motifs in a module are combined, and how the score of multiple binding sites for the same factor is calculated.

As discussed e.g. by Tompa *et al*. [10] it is very difficult to compare the performance of methods, in particular on complex genomes like the human. Furthermore, methods will also differ in aspects like average running time, need for manual parameter-tuning, exhaustiveness of results, general usability and so on. Individual methods may also perform better on one type of genomes compared to others, making it difficult to compare performance on a general scale. We have therefore to a large extent deliberately avoided comparing relative performance of individual methods. We mainly indicate important elements of the problem, and show the breadth of possible solutions that have been tested, both when it comes to established elements of motif discovery, such as single motif models, as well as less common approaches, such as the incorporation of DNA structure. However, there is a definite need for more standardized routines for testing and comparing alternative approaches to motif discovery, and the work by Tompa *et al*. [10] is an important step in that direction.

## Biological background

The system for transcriptional regulation of the eukaryotic genome is complex. The regulatory processes are found at several hierarchical levels, in particular at the sequence level, the chromatin level and the nuclear level [11]. The sequence level includes coding regions, regulatory binding sites and sequence elements affecting the 3-dimensional fold of the chromatin fiber. It is mainly the binding sites for transcription factors that will be discussed here.

In eukaryotic cells DNA is packed as chromatin, and this affects transcriptional regulation. The basic unit consists of 150 base pairs of DNA wrapped 1.7 times around a protein octamer, consisting of histones. This unit is called the nucleosome, and it can exist in different structural and functional states. Transitions between states are linked to gene activity. These transitions are influenced by post-translational modifications of histones, and this is often described as the histone code. Also gene silencing by DNA methylation is an important chromatin modification.

In addition to the linear (sequence) and pseudo-linear (chromatin) organization of DNA, it is also organized in a highly folded state. This brings together genome regions that are far apart, which may affect the co-regulation of these regions. However, we lack efficient tools for studying global chromatin folding.

In particular the transcriptional regulation at the sequence level has been extensively studied, and several reviews are available, e.g. by Werner [12], Wray *et al*. [13] and Pedersen *et al*. [14]. The key regulatory region is the promoter region, located upstream of the coding sequence. It is often separated into the basal (or core) promoter, where the transcriptional machinery is assembled, and the general promoter, where most of the transcription factors bind. The promoter basically integrates information about the status of the cell, and adjusts the transcription level according to this information. The transcription factors are proteins that bind to specific DNA motifs. These motifs are short. The effective length may be just 4–6 base pairs (bp) for a typical binding site, although the region affected by the transcription factor (the footprint) is longer, typically 10–20 bp. Each gene contains a large number of binding sites, 10–50 binding-sites for 5–15 different transcription factors is not unusual. These transcription factor binding sites are often organized in modules consisting of several binding sites, where each module produces a discrete aspect of the total transcription profile. For many genes most of the binding sites are found within a few kb upstream of the start site. However, the variation is large, the size of the region where cis-regulatory elements are found can vary by nearly three orders of magnitude from a few hundred bp to >100 kb. Regulatory regions have also been found downstream, in introns and even in exons of genes. The actual transcriptional regulation is achieved through a complex, combinatorial set of interactions between transcription factors at their binding sites [15].

## An integrated framework

As motif discovery methods can be very complex, with many possible differences, several authors have proposed frameworks for classifying motif discovery methods. Brazma *et al*. [16] categorize motif discovery methods with respect to whether they use explicit negative sequence sets or not, expressiveness of the pattern models, whether patterns are deterministic or statistical, and whether the algorithms are pattern driven or sequence driven. In a later paper Brazma *et al*. [17] define a three step paradigm consisting of choosing a class of grammars (motif model), designing a rating function (motif score), and developing an algorithm. However, the major recent advances in the field have been on modeling of regulatory regions, rather than individual sites, and on integration of additional data. The frameworks mentioned above are not well suited to highlight developments in these directions. We therefore use an extended, integrated framework for the description of motif discovery methods, where both the representation of the transcription factor based regulatory system itself, as well as additional sources of information, can be represented.

The most basic level of our framework (Level 1) represents the binding of transcription factors (TFs) to short contiguous sequence segments. These sequence segments are modeled by single motif models that give a distinct score for each sequence segment in a regulatory region. This score is based on the match between the sequence segment and a motif consensus model, and on the prior belief that any regulatory element may occur at the given location.

The next level of our framework (Level 2) represents modules: clusters of TFs that bind to DNA in proximity to each other, but with a certain flexibility regarding distance between binding sites. This is modeled by a composite motif model, consisting of a set of single motifs. Given a set of positions, one for each single motif, the score of a composite motif can be calculated from the score of single motifs at given positions as well as inter-motif distances.

The third level of the framework (Level 3) represents how several modules may act together, possibly in a combinatorial manner, to determine the regulation of a single gene. This is modeled by a gene score function that combines composite motif scores across the regulatory reglon(s).

The final level of our framework (Level 4) represents several sets of modules acting on sets of genes, e.g. at the genome level. Scores at this level are mostly used for evaluation and ranking of *de novo *discovered motifs. The evaluation is based either on overrepresentation of motifs, or on correspondence between motif scores and experimental data.

A schematic view of our framework, reflecting the different levels of regulatory processes, is given in Figure

1. The different elements of this figure will be described in more detail in the following sections.

We will now use this framework to categorize a large number of existing methods for motif discovery. Table 1 gives an overview of how various elements of our framework are approached by selected methods, including both novel and more established approaches. A larger table, which includes most current methods, is available as supplementary material [18].

**Table 1:** Overview of methods. The match model is the consensus representation of a single motif, motif combination is how the component scores of a composite motif are combined, and distance score is how the conservation of inter-motif distances within a composite motif is modeled.

**ALGORITHM NAME**	**MATCH MODEL**	**MOTIF COMBINATION**	**DISTANCE SCORE**
Weeder [42]	mismatch	-	-
Dyad analysis [35]	oligos	dyad^1^	constraint
MCAST [71]	PWM	sum	gap penalty
REDUCE [67]	PWM	dyad	constraint^2^
MDScan [87]	PWM	-	-
Gibbs sampler [97]	PWM	intersection^3^	uniform
MEME [98]	PWM	-	-
LOGOS [73]	DM	HMM	distribution
Motif regressor [89]	PWM	-	-
ModuleSearcher [70]	PWM	sum	window^4^
Stubb [48]	PWM	HMM	window
GANN [60]	flexible	ANN^5^	window
ANN-Spec [86]	PWM	-	-
(Wasserman) [58]	PWM	Logistic regr.	window
CoBind [68]	PWM	sum	window
Cister [72]	PWM	HMM	distribution
SeSiMCMC [122]	PWM	-	-
SMILE [40, 123]	mismatch	intersection	constraint
BioProspector [49]	PWM	sum	constraint
(Segal) [94]	PWM	-	-
(Sinha) [33]	reg.exp	dyad	constraint
ConsecID [56]	PWM	intersection	window
SCORE [69]	IUPAC	intersection	window
Gibbs recursive [52]	PWM	mixture model	distribution
(Hong) [95]	PWM	-	-
AlignACE [124]	PWM	-	-
Improbizer [117]	PWM	-	-
CisModule [119]	PWM	mixture model	mixture model
(Thompson) [66]	PWM	Markov model	constraint

^1^Two single motifs that both have to occur

^2^Separate constraints on each inter-motif distance

^3^Several single motifs that all have to occur

^4^All single motifs have to occur within a sequence window of restricted length

^5^Artificial neural network

## Single motif models (Level 1)

Transcription factors bind to specific short segments of DNA, transcription factor binding sites. This is the most basic element of the regulatory system, and can be modeled using single motif models. *A single motif model is defined as a function m*_*g *_: ℕ→ℝ *that maps a sequence position p as a non-negative integer to a real numbered motif score m*_*g*_(*p*). It consists of a match score m*(*p*) *and an occurrence prior o*_*g*_(*p*).

The function *m*_*g*_(*p*) returns a value indicating whether an occurrence of the motif is found at position *p*. This function is typically the product or sum of two conceptually different functions. The match model, m*(*p*) gives the degree of match between the substring beginning at position *p *and an underlying consensus model. The occurrence prior, *o*_*g*_(*p*), gives the prior belief that position *p *represents a regulatory element for gene *g*.

### Match models

In the most general sense, the match model m*(*p*) is a function that gives a distinct score for any given substring. However, the number of free parameters has to be restricted to allow training of the model from a limited number of examples (e.g. known regulatory elements). Numerous match models have been proposed, and they are often divided into two groups, deterministic models with binary scores and probabilistic models with weighted scores.

#### Probabilistic match models

The most widely used probabilistic model is without doubt the position weight matrix (PWM), also known as position specific scoring matrix (PSSM), that assumes independence between positions [3]. The score of an aligned substring is the log-likelihood of the substring under a product multinomial distribution. PWM scores can also be described in a physical framework as the sum of binding energies for all nucleotides aligned with the PWM [19].

Many different extensions to the basic PWMs have been proposed in the literature. Most of these extensions concern positional dependencies within a motif. There is an ongoing discussion on the importance of such positional dependencies, see for instance [20-22].

The most direct way of incorporating dependencies within motifs is to extend the PWM to include pairs of correlated positions [21,23]. Another straightforward approach is to use a mixture model in which the motif occurs as one of a limited number of stochastic prototypes [24]. Each stochastic prototype may be a traditional PWM, or any other model discussed in this section. A third extension is to model probabilistic motifs as n'th order Markov chains [25]. However, it is hard to find a good compromise between a high n that may give too many free parameters and a low n that may miss out the dependencies of interest. If the relative importance of dependencies varies within a motif, a variable-length Markov model (VLMM) [26] may be preferable. Furthermore, if some long-range dependencies seem to be significantly stronger than dependencies between neighboring positions, the order of the positions in the Markov chain may also be permuted before a VLMM is applied [27].

Another way to model dependencies is to use Bayesian networks. Barash *et al*. [24] discuss different Bayesian network models and conclude that the use of a Bayesian tree model, or possibly a mixture of trees, is a good compromise between the number of free parameters, the ability to model dependencies, and computational tractability. Similarly, Ben-Gal *et al*. [28] argue for variable order Bayesian nets.

Instead of focusing on dependencies between specific nucleotides at different positions, Xing *et al*. [29] model the distribution of conserved positions within a motif. In this model there is an underlying Markov chain of position prototypes. Each prototype defines a certain Dirichlet distribution on the parameters of the multinomial nucleotide distribution at that position. The underlying Markov chain favors transitions between position prototypes with similar degrees of conservation. This makes it possible to favor models where highly conserved positions are partially contiguous rather than evenly spread out in the motif. The work of Kechris *et al*. [30] achieves similar properties by assigning conservation types (strong, moderate or low) to blocks of motif positions.

#### Deterministic match models

A deterministic match model evaluates to a binary value indicating either hit or no-hit. The three main kinds of deterministic match models are oligos, regular expressions and mismatch expressions.

The simplest deterministic model is the oligo model. This is a function that is 1 for a single specific substring, and 0 for all other substrings. The oligo model was commonly used in early motif discovery methods, but has also been used in recent word-counting methods [31-33] and dictionary models [34].

A regular expression model m*(*p*) returns 1 if the given substring is matched by an underlying regular expression. As reviewed by Brazma *et al*. [16], the models used in motif discovery are typically composed of exact symbols, ambiguous symbols, fixed gaps and/or flexible gaps. Regular expression models are used in e.g. [33,35-38].

Many methods use mismatch expressions as motif match models, e.g. [39-44]. These models evaluate to 1 if the number of mismatches (Hamming distance) between a substring and the underlying consensus substring is below a given threshold. A variant is described in [45], where the threshold is on the sum of mismatches between all motif occurrences and the underlying motif substring. A similar variant, with a threshold on mismatches between occurrences in sequences arranged in a phylogenetic tree, is described in [46].

The probabilistic models are much more expressive than the deterministic models. In fact, all oligos, regular expressions and mismatch expressions can be represented as PWMs. However, a major benefit of the deterministic models is that they often allow exhaustive discovery of optimal motifs.

### Occurrence priors

The genetic context of a regulatory element is important for its activity. Distance to transcription start site, sequence conservation in orthologous genes, DNA structure and presence of CpG-islands may be relevant factors. In our model, these context features are represented by an occurrence prior, *o*_*g*_(*p*), representing the prior belief that an (unspecified) regulatory element is located at a given position *p*.

The simplest kind of occurrence prior is a motif abundance ratio [47]. This ratio influences only the number of substrings that count as occurrences. Another simple prior is strand bias, which corresponds to an occurrence prior that is higher on one strand than on the other [48]. Several methods including Bioprospector [49] and TFBScluster [50] optionally constrain the search to only one of the strands, which corresponds to a binary strand bias.

#### Spatial distribution of binding sites

In higher organisms, regulatory elements may be located far upstream of the gene, downstream of the gene, in introns, and even in exons. Nevertheless, most known elements are located immediately upstream of the transcription start site (TSS). In general, this can be represented by a function giving the prior belief that a regulatory element is located at a given position relative to the TSS. An occurrence prior based on the empirical distribution of element locations in *E. coli *has been used in [51] and [52]. Nevertheless, the by far most common approach is to only search for motifs in a fixed region upstream of TSS, which corresponds to a binary function for *o*_*g*_(*p*).

#### Conservation in orthologous sequences

The term phylogenetic footprinting is commonly used to describe phylogenetic comparisons that reveal conserved elements in regulatory regions of homologous (in particular orthologous) genes [53].

The reasoning behind phylogenetic footprinting is that since regulatory elements are functionally important and are under evolutionary selection, they should evolve much more slowly than other non-coding sequences. Moreover, genome-wide sequence comparisons and studies of individual genes have confirmed that regulatory elements are indeed conserved between related species [54]. More specifically, Krivan and Wasserman [55] reported that highly conserved regions were around 320 times more likely to contain regulatory elements than non-conserved regions, based on findings from a set of liver-specific genes.

Several methods exploit information about conservation in orthologous gene regulatory regions by searching for motifs only in highly conserved sequence parts (typically human-mouse orthologs) [44,48,56,57]. This approach corresponds to using a binary occurrence prior that is 1 if the conservation score is above a given threshold and 0 otherwise. Wasserman and Fickett [58] use non-binary conservation scores, but they do not incorporate these into the search as priors. Instead, they use conservation to filter the discovered motifs. Similarly, Xie *et al*. [38] calculates the proportion of motif occurrences that are conserved in related species, and uses this in the evaluation of motif significance. Finally, Wang and Stormo [59] constructs phylogenetic profiles, representing the frequency of nucleotides in each position based on multiple alignment of promoters in related species.

#### DNA structure

The three-dimensional structure of DNA, densely packed as chromatin, inhibits transcriptional initiation *in vivo *[14]. The bendability of a region, as well as its position in DNA loops, may indicate whether it contains regulatory elements or not. Only a few motif discovery methods take DNA structure into consideration. Beiko and Charlebois [60] average structure scores of all k-mers in a window around a given position, independently of any particular motif. Conversely, Pudimat *et al*. [61] incorporate helical parameter features [62,63] in a Bayesian net that is specific for each motif.

#### Nudeotide distribution

Both high GC content and presence of CpG-islands may indicate that a region contains regulatory elements. The method of Pudimat *et al*. [61] is one of a few methods that take GC content and CpG-islands into consideration when calculating motif scores.

## Composite motif models (Level 2)

Clusters of binding sites for cooperating TFs, often called modules, are believed to be essential building blocks of the regulatory machinery. Werner [12] states that "Within a promoter module, both sequential order and distance can be crucial for function, indicating that these modules may be the critical determinants of a promoter rather than individual binding sites". The multitude of models developed for the discovery of modules is another indication of the conceived importance of this. It is therefore natural to define a computational motif model that represents a combination of single motifs.

*A composite motif model is defined as a function c*_*g*_: 2^N^→ℝ *that maps a set of single motif sequence positions ** as non-negative integers to a real numbered composite motif score c*_*g*_()*. It consists of single motifs *_*g*_.

The function *c*_*g*_() consists of a set of (generally different) single motifs _*g*_, with each single motif contributing with a separate score at its position. In addition, functions may be defined on the distances between single motifs. Given a set of positions, the score of a composite motif will typically be the sum or product of individual single motif and distance scores.

### Distance functions

Many different models have been proposed to capture the importance of inter-motif distances within a module. Several methods put constraints on the distances between consecutive motifs, requiring either fixed distances [33,49], distances below thresholds [64-66], or distances within intervals (e.g. [33,35,43,49,67]).

Another common way of capturing the importance of proximity is to constrain all single motifs to be within a window of a certain length (e.g. [48,58,68-70]). This corresponds to a threshold on the maximum distance between any two single motifs. A more general approach is to define non-binary score functions on the distances between single motifs. This can simply be functions that increase linearly with distance as in [71]. Similarly, a geometric distribution on inter-motif distances follows implicitly from many HMM models [72,73], and is assumed explicitly in Gupta and Liu [74].

The conservation of inter-motif distances across modules can also serve as a basis for distance score functions. Wagner [75] calculates a distance score from the *p*-value of observing the given degree of distance conservation in a background model of Poisson-distributed inter-motif distances. Similarly, Frech and Werner [76] calculate scores by comparing the distances with a histogram of distances between the same regulatory elements in other modules.

We have implicitly assumed in this discussion that distance is the number of base pairs between two positions in the genome. It is in principle possible to measure distance in other ways. An example is to require all motifs in a module to be on the same strand [36], which corresponds to a simple binary distance function. More importantly, as our understanding of DNA folding increases, new and more complex distance measures may appear.

### Combining single motifs

There are many ways in which a set of single motif and distance scores can be combined into a single measure.

For methods using deterministic match models and constraints on distances, all component scores are binary. Furthermore, many probabilistic methods use thresholds on single motif scores to obtain only binary values. The composite motif score is then typically the intersection of component scores (e.g. [56,75,77,78]). A variation of this is to require that *M *out of *N *single motif scores are 1 [79]. Similarly, the count of binary single motif values can be used directly as a composite motif score [33,80,81].

For methods that use non-binary single motif scores, a common approach is to calculate the sum of single motif and distance scores [71,76]. Some methods require that all distance functions are 1, and if they are, composite motif score is the sum of single motif scores [68,70,82,83].Similarly, the method Modulescanner sums only single motif scores above a threshold, and MotifLocator sums the *N *highest single motif scores [70]. Another variation is to multiply the sum of single motif scores with a motif density factor, calculated from the length of the window that contains all the single motifs [64]. Finally, a few methods take the composite motif score to be the highest single motif score [42], or the lowest single motif score [84].

Many specialized models have also been used to combine single motif and distance scores, e.g. the hidden Markov model (HMM) [73], history-conscious HMM (hcHMM) [48], self-organizing map (SOM) [85], and artificial neural network (ANN) [60]. In all of these models, the score of several homotypic and/or heterotypic single motifs are combined in a relatively complex way.

## Gene level models (Level 3)

In addition to the motif scores, which are defined for specific positions, we may also be interested in the presence of motifs across the regulatory regions of a gene. The possibility of multiple binding sites for TFs is often not discussed explicitly in articles presenting motif discovery methods. Scores at this level may, however, be relevant both when predicting which genes are regulated by a TF or module, and when evaluating the significance of a *de novo *discovered motif.

*A gene score model is defined as a function G*_*c*_: ℕ→ℝ *that maps a gene index g as a non-negative integer to a real numbered gene score G*_*c*_(*g*)*. It consists of composite motif models c*_*g*_().

The gene level score is calculated from composite motif scores, *c*_*g*_(), across the regulatory region of gene *g*, and is referred to as gene score. For methods that only discover binding sites for single TFs, the composite motif score is simply the single motif score.

### Multiple binding sites

The gene level score is often defined simply as the maximum motif score in the regulatory region(s) of a gene [46,70,81,86,87]. This corresponds to an implicit assumption of exactly one relevant occurrence of a motif in the regulatory reglon(s).

It is, however, reasonable to assume that the presence of multiple binding sites for TFs plays an important biological role that should not be neglected. Many methods therefore calculate gene score from all motif scores across the regulatory region(s) of a gene. As motif scores are typically log-scores, most methods add the exponentials of motif scores (e.g. [67,68,88-90]). A slight variation is to only sum motif scores above a certain threshold [71].

In addition to these approaches, many variations have been used to calculate gene score. Caselle *et al*. [91] and Cora *et al*. [57,92] calculate gene score as the *p-*value of the observed set of motif scores. Curran *et al*. [93] calculate gene scores based on logistic regression. Similarly Segal *et al*. [94] use a logistic function, and Hong *et al*. [95] a hyperbolic tangent, on the sum of motif scores. Finally, Beiko *et al*. [60] use an artificial neural network to combine motif scores.

The dictionary models of Bussemaker *et al*. [34] and Gupta and Liu [96] represent a special case, as they always span whole regulatory regions. In these methods the score of all valid segmentations of the region into contiguous words from the dictionary is added together to form the gene score.

### Multiple modules

In addition to multiple binding sites for the same module, a set of different modules may also be introduced at the gene level. A gene may be seen as having several regulatory regions, with tight distance constraints between binding sites within a regulatory region (module), and larger and more variable distances between different regulatory regions. Xing *et al*. [73] define an HMM that can represent different modules of binding sites with different implicit geometric distributions within and between modules. This model can also represent different intra-module background distributions in addition to the global inter-module background distribution. This corresponds to a gene score that is calculated from the scores of several different composite motifs across the regulatory regions of a gene.

## Genome level models (Level 4)

Motif scores at the genome level are generally used for significance evaluation of *de novo *motifs, although it may in some situations also be relevant to look at the presence of motifs (TFs or modules) in different genomes. Here we focus on the first situation, evaluation of motif significance at the genome level. In most cases the genome level score is based on just the (assumed) regulatory regions for a selected subset of the genes.

*A genome score model is defined as a function s*_*c*,*F *_: ℕ→ℝ *that maps a genome index i as a non-negative integer to a real numbered genome score s*_*c*,*F*_(*i*)*. It consists of a gene score model G*_*c*_(*g*) *and a gene membership function μF*(*g*).

Genome score (motif significance) is typically based on either the genome level overrepresentation of the motif, or on the correspondence between gene scores and experimental data.

### Motif overrepresentation

Computational motif discovery is possible primarily because motifs representing regulatory motifs are overrepresented. Many methods use this overrepresentation directly when evaluating the significance of a discovered motif. The exact way of calculating motif significance varies from method to method, but can roughly be divided into five different approaches.

The most direct approach is to determine overrepresentation by comparing observed motif scores with expected scores from a background model. More specifically, the *p-*value [37,69] and *z*-score [33,39] of the observed sum of gene scores has been used. The background is typically a higher order Markov model, with parameters estimated from the sequences used for motif discovery. Shuffled control sequences may also be used as background [97].

A simpler approach is to compare only the raw sum of gene scores when ranking motifs. This is equivalent to the first approach under the assumption of equal expected scores for all motifs in the background model.

A third approach is to use a significance measure related to the information content (IC) of discovered PWMs [98]. For methods that use mixture models of log-ratio PWMs and background, the PWM with highest IC corresponds to a maximum likelihood solution of the mixture model.

A common approach in deterministic motif discovery is to calculate two separate values when evaluating motifs, one concerning the support, or coverage, of a motif, and a second concerning the unexpectedness of a motif [40,99,100].

The fifth approach is completely different, and focuses only on overrepresentation of motif combinations. Motif significance is based on the observed versus expected scores of *composite *motifs, given the observed score distribution of *single *motifs. The significance can for instance be the *p*-value of the observed composite motif scores in a background model where all single motif occurrences are randomly reshuffled [56].

### Correspondence with experimental data

In recent years, the development of microarray technology has revolutionized studies of regulatory processes, in particular because it can be used to identify genes that are co-regulated under specific conditions. Microarrays are used to measure relative expression levels of genes in a set of experiments. This may be e.g. time series experiments like cell cycle studies or before/after experiments like stress response studies and studies of malignant vs. normal tissue. It is a reasonable hypothesis that genes showing synchronized changes in expression levels share important aspects of transcriptional regulation, e.g. transcription factor binding sites. Sets of genes showing co-regulation may therefore be used for data mining for shared regulatory motifs [101], although it has been shown that this type of data mining is difficult and error prone [10]. A variant of this approach is to cluster genes based on expression similarity with specific transcription factors [102,103].

Recently, genome-wide binding analysis like ChIP/chip experiments have appeared as an approach for more reliable identification of actual binding site regions [104,105]. In a ChlP/chip experiment a known transcription regulator is tagged with an antibody epitope, and the tagged regulator is expressed in a suitable system where it binds to DNA, either directly or via other proteins. The complex is then chemically crosslinked, the DNA is fragmented, and the protein/DNA complex is isolated by immunoprecipitation. The genomic position of the DNA fragment is then identified by a microarray experiment. This gives the location of binding sites for this specific regulator, although the relevance of the information may be limited by the specific set of experimental conditions used and the resolution of the experiment itself (DNA fragment size and genome resolution on the microarray chip).

Besides ChIP/chip and microarray experiments, gene groups are often formed from conserved orthologous genes [46,88,106,107], or genes with similarities in functional annotation [32,57]. Finally, genes that make up functional pathways, genes that are homologous to regulons from a well-studied species, and groups of genes derived from conserved operons have also been used [108].

Many methods cluster genes based on experimental similarities, assigning each gene to a single group of putatively co-regulated genes. All genes are then treated equally during motif discovery, regardless of the degree of similarity between a gene and the rest of the group (e.g. [66,93,95,108,109]). However, as a gene may be co-regulated with several groups of genes, depending on conditions, it may make sense to use fuzzy sets to represent prior grouping of genes. In our model, every gene *g *has a weighted membership *μF*(*g*) in each fuzzy set *F*. Segal *et al*. [81] and Liu *et al*. [87] are among the few authors that have used weighted values for set membership during motif discovery.

The correspondence between gene level scores and experimental data may be used as a measure of motif significance. This can be calculated in several ways. One approach is to evaluate the fit of a logistic regression from gene scores *G*_*c*_(*g*) to membership values *μF*(*g*) [58,93]. A simplification of this approach is to compare binary gene scores with binary membership values, and calculate the mismatch ratio [95] or ROC_50 _score [71]. Alternatively, grouping of genes can be avoided altogether, and motif significance can be measured as the fit of a linear regression directly from gene scores to observed log-expression in microarray experiments [67,89,94].

Park *et al*. [110] consider the problem in the opposite direction. They first discover motifs in the regulatory regions of all genes and form groups of genes that share common motifs. Motif significance is then measured as the similarity in gene expression within the group formed from the common motif.

Finally, Holmes and Bruno [111] calculate the joint likelihood of both shared motifs and expression similarity for hypothesized gene groups.

Although several methods may be configured to use different kinds of experimental data [32,57,108], only a few methods try to combine different kinds of data in a single similarity measure. Takusagawa and Gifford [37] use the GRAM algorithm [112] to cluster genes based on both ChIP-data and gene expression data. Further work incorporating more kinds of experimental data and using fuzzy set membership could give more robust priors on co-regulation and increase the sensitivity of motif discovery.

## Some algorithmic concerns

An important trade-off in motif discovery is between representational expressibility and computational efficiency. For the case of binary priors and restricted deterministic motif models, several algorithms exist that can exhaustively discover the optimal motifs [99,100,113].

However, probabilistic motif discovery algorithms do not guarantee returning the global optimum when applied to realistic problems. These algorithms are typically based either on iterative refinement or stochastic optimization. Expectation maximization (EM) [98,114-117] is the most widely used iterative refinement method, but variational EM [73] has also been used. The stochastic optimization technique most widely used for motif discovery is Gibbs sampling [49,52,97,118], sometimes combined with general Metropolis-Hastings [47,96,119]. Recently, genetic algorithms [82], evolutionary Monte Carlo [74] and simulated annealing [27,81,120] has also gained some popularity.

Seed-driven algorithms have been used with success in deterministic motif discovery. They start by evaluating seeds from a very restricted class of simple motifs, and then expand promising seeds to full motifs either heuristically [121] or exhaustively [100]. A promising approach to motif discovery is first to use efficient deterministic motif discovery, and then use the highest scoring deterministic motifs as seeds for probabilistic motif discovery with expressive models. In addition, motifs may first be discovered in the sequence parts with highest priors, and then be used as seeds for motif discovery in the full set of sequences. The method of Liu *et al*. [87] is a good example of such a strategy. Several overrepresented mismatch expressions are first discovered in upstream regions of the genes with highest group membership (*μF*(*g*)). The highest scoring mismatch expressions are then used as seeds for probabilistic motif discovery in the whole set of sequences.

## Comparison of methods

Given the very large number of different methods for motif discovery, it is obviously crucial to have good test methods in order to identify the most promising approaches. However, this has turned out to be a challenging problem by itself.

It is difficult to identify optimal test sets for benchmarking. When comparing the performance of methods the output has to be compared against some biological truth. Even though biological sequences with experimentally verified binding sites are available, they may contain additional (yet unidentified) binding sites that may show up as false positives in motif discovery. Using implanted motifs in synthetic background sequences may avoid this problem, but creates new problems with respect to realistic background sequences and motif distributions, in particular for composite motifs. It may also be difficult to get enough data to get a good representation of the diversity of regulatory regions.

It is also difficult to know whether a test result actually reflects the assumed methodological difference between alternative approaches. Many methods will require different degrees of parameter tuning. This may introduce bias in test results, and makes automatic testing difficult. Typical examples of tunable parameters may be motif length, expected number of motif occurrences, and inter-motif distances. Also, many methods make use of additional data, in addition to the actual sequences, in order to increase performance. For instance, several methods include phylogenetic footprinting using related organisms. Finally, different implementations may have been optimized and fine tuned to different degree. This makes it difficult to distinguish between the performance of underlying algorithmic approaches and the effect of several years of tweaking on a specific implementation. If radically different and possibly better performing approaches are to be identified, it is essential that novel algorithmic approaches are tested against existing methods in comparable frameworks and implementations.

These challenges make it difficult to actively compare the performance of alternative approaches and use this as a basis for recommendations. The seminal benchmark of single motif discovery methods by Tompa *et al*. [10] mainly concludes that biologists are advised to use a few complementary tools in combination rather than relying on a single one, and to pursue the top few predicted motifs of each rather than the single most significant motif of any given method. Some of the most established methods, such as MEME, AlignACE and ANN-Spec, performed reasonably well, at least on simple data (e.g. yeast). However, the best method overall on these datasets was the more recent method Weeder. Only single motif discovery was tested in this work. No other study of comparable breadth has tested composite motif discovery methods, probably because it is even more challenging to find suitable test sets and to evaluate alternative methods for composite motifs.

However, on a more general basis we believe that some recent developments on expressive models for combination of motifs are particularly interesting. The method "motif regressor" represents a relatively simple, yet promising approach [89]. First it uses the MDScan algorithm [87] to discover single motifs based on CHiP-chip data. Motifs that are too similar to the background distribution are filtered out, and the remaining motifs are used as features in a multiple regression from gene level scores of motifs to gene expression levels. In this way, only motifs that serve (independent) explanatory roles on gene expression are retained. Another interesting approach is the LOGOS method [73] that uses a hidden Markov model (HMM) to model the combinatorial nature of binding sites. Furthermore, single motifs are modeled by a HMDM model [29] that promotes binding sites with certain spatial distributions on single nucleotide conservation. All of this is combined using a coherent probabilistic model.

## Conclusion

The field of motif discovery brings together researchers from several disciplines, in particular from biology, statistics and informatics. Additionally, research in the field is fairly recent and moving at a fast pace. This has resulted in a broad range of computational methods that are described with different vocabulary and different focus, making it difficult to spot similarities as well as differences between methods. Most papers on novel computational methods tend to focus on the authors' own data sets and scientific problems. Hence, the authors often put less emphasis on giving a clear description of the algorithm itself, e.g. precisely what it requires as input, how it evaluates motifs, and what it returns as output. This makes it harder to compare methods based on their descriptions.

When trying to compare the accuracy and computational efficiency of methods by measurement, there are additional problems. The choice of data set, choice of performance measures and tuning of program parameters all have strong influence on the relative performance of methods [10].

Establishing a standardized framework for testing would be an important contribution to the field. Such a framework should include a collection of diverse data sets and several complementary measures of performance. Furthermore, a consensus on what constitutes essential aspects of motif discovery methods could ease the comparison of methods, making it easier to choose between or integrate different approaches. This could also make it easier for researchers to identify the choices that have to be made when a new model or approach is being developed, as well potential previous models where these choices already have been evaluated. The integrated model described in this paper may be one step towards a common vocabulary and framework for this problem.

When surveying recent literature we have made several interesting observations. One is the sheer breadth of approaches used in the field when it comes to how motifs are modeled and how experimental information is integrated. A somewhat related observation is the great variation between motif models, even when it comes to aspects that are typically not discussed explicitly in papers, e.g. how the gene level score is calculated. In other words, some papers implicitly treat the chosen model as obvious and the only possible solution, whereas comparison to similar methods shows that there indeed are several possible approaches that should have been evaluated.

A third observation is that even though there are many aspects of a basic motif model that can be improved, each article typically considers only one of them. If we add together the possible enhancements to different parts of the models for regulatory regions, and the different kinds of additional data that have been incorporated, based on all papers in the field, wee see a much more complex and enhanced model. Although such a model may be too complex for a full implementation, one should at least make deliberate choices with respect to which elements are included in a given approach. Hopefully the integration of techniques and experiences across existing approaches will give rise to refined and advanced methods with higher sensitivity than what we have seen so far.

## Reviewers' comments

### Reviewer's report 1

Eugene V. Koonin, National Institutes of Health, Bethesda, MD, USA

This is a detailed and useful survey of the computational approaches used for discovery of sequence motifs in DNA, with an emphasis on transcription-factor-binding sites. The paper is well-structured and properly referenced. I believe that many researchers will find it helpful.

### Reviewer's report 2

Philipp Bucher, Swiss Institute of Bioinformatics and Swiss Institute for Experimental Cancer Research, Switzerland (nominated by Mikhail Gelfand, Institute of Information Transfer Problems, Moscow, Russia)

This article clearly responds to a need. The literature on motif discovery methods has grown vast, confronting the reader with a bewildering variety of methods and concepts. The authors rightly point out that the different methods are not always appropriately described in the scientific articles. Underlying assumptions are often not explicitly stated, and methodological choices are not mentioned as they may appear self-explanatory to the developers.

This comprehensive review makes and attempt to consolidate the field by providing a framework for categorizing the large number of existing motif discovery methods. The various methods are classified according to four hierarchical levels of genome organization: Individual motifs, composite elements, genes, and genomes. This framework is useful from a biological perspective as it allows for joint presentation and comparison of methods that address similar questions. A potential drawback is that technical issues may be arbitrarily spread over different parts of the manuscript. For instance, it is debatable whether the significance measure related to the information content of a PWM, which is used by MEME, should be presented under the heading " genome level models".

What is lacking in this review is a historical perspective. The manuscript focuses on recent work disregarding largely how current concepts have evolved over time. I would propose to add some of the earlier landmark papers to the bibliography, for instance:

Korn LJ, Queen CL, Wegman MN. (1977) Computer analysis of nucleic acid regulatory sequences. Proc Natl Acad Sci USA. 10:4401–4405. This is perhaps the first paper describing a computer algorithm that helps to find an over-represented sequence motif.

Queen C, Wegman MN, Korn LJ. (1982) Improvements to a program for DNA analysis: a procedure to find homologies among many sequences. Nucleic Acids Res. 10:449–456. Perhaps the first paper implicitly using a mismatch model for motif discovery. It also presents an efficient algorithm to find optimal motifs of this type.

Staden R. (1984) Computer methods to locate signals in nucleic acid sequences. Nucleic Acids Res. 12:505–19. First paper proposing PWMs with weights proportional to the logarithms of the observed base frequencies.

Brendel V, Trifonov EN. (1984) A computer algorithm for testing potential prokaryotic terminators. Nucleic Acids Res. 12:4411–4427. This work extends position independent weight matrices to dinucleotide matrices, thereby accounting for nearest-neighbor dependencies.

Galas DJ, Eggert M, Waterman MS. (1985) Rigorous pattern-recognition methods for DNA sequence sequence analysis of promoter sequences from Escherichia coli. J. Mol. Biol. 186:117–128. An early paper presenting a method that takes into account a motif's distance to the transcription start site.

Berg OG, von Hippel PH (1987) Selection of DNA binding sites by regulatory proteins, statistical-mechanical theory and application to operators and promoters. J. Mol. Biol. 193: 723–750. Provides a physical (thermodynamic) interpretation of PWMs.

**Author response: ***We have added a brief historical overview to the introduction, including most of the references mentioned here.*

Regarding present-day genome-wide approaches, the following two papers may be worthwhile to mention: Xie X, Lu J, Kulbokas EJ, Golub TR, Mootha V, Lindblad-Toh K, Lander ES, Kellis M. (2005) Systematic discovery of regulatory motifs in human promoters and 3' UTRs by comparison of several mammals. Nature. 434:338–345.

Wang T, Stormo GD. (2005) Identifying the conserved network of cis-regulatory sites of a eukaryotic genome. Proc Natl Acad Sci USA. 102:17400–17405. Epub 2005 Nov 21.

**Author response: ***These references have been added to the article.*

### Reviewer's report 3

Frank Eisenhaber, Institute of Molecular Pathology, Vienna, Austria

The question on how to determine the occurrence of regulatory elements in nucleic acid sequences is in the center of biomolecular sequence analysis since many decades. The literature has become large, it is not easy to oversee and to evaluate. Thus, a review in this area is appropriate.

The present revised MS of Sandve and Drablos has an acceptable style and language, the article is well structured and easy to read.

The authors wish to present their quite formalized, integrated framework (level 1 – small motif binding sites, level 2 – clusters of sites in close proximity (= modules), level 3 – combinations of modules in the regulatory region of a gene, level 4 – sets of modules in regulatory regions of sets of genes) for organizing the vast literature and for delineating the elementary recognition tasks in the prediction of regulatory elements.

From the very beginning (last paragraph in the introduction), the authors refrain from a comparison of various methods with respect to their performance. Moreover, there is no quantitative assessment in the manuscript that allows to estimate what can be expected from the group of methods described in this review in general. It is the pity reality that prediction of regulatory regions is pretty unreliable with both false-positive and false-negative prediction rivalling the number of true predictions.

The following manuscript text is merely a compilation of the variations in mathematical formulations used in the different methods in the literature. For assessing the relative merit of the various approaches, the authors do not have appropriate criteria. Although a performance comparison is difficult and gold standard test sets are not readily available, it would nevertheless give some hint on the reliability of methods and their relative accuracy. The comparative work of Bajic VB, Tan SL, Suzuki Y, Sugano S. (Promoter prediction analysis on the whole human genome. Nat Biotechnol. 2004 Nov;22(11):1467–73) is focused on a very specifc type of a regulatory region but it is at least a beginning of a large-scale performance evaluation. If the authors do not wish to get involved in such a comparative study, they should at least provide a review of published data. To a certain extent, this has been provided in an additional section in the revised version but the wording appears very polite and a quantification of performance is not provided. To emphasize the view of a practitioner, this is what matters.

**Author response: ***We acknowledge the concern about evaluation of methods, which is why we have included an expanded section in the revised version discussing comparison of motif discovery methods. However, we do not feel that it is currently possible to give clear recommendations on the issues considered in our survey. We have elaborated more on the reasons for this in our revised manuscript. As our focus is on the recent development of methods taking combinatorial mechanisms and additional data into consideration, the benchmark of Tompa et al. (2005) could only give limited guidance. The recent article of Bajic et al. is also very interesting, but it considers methods for promoter prediction and in particular prediction of transcription start sites *(*TSS*)*. These methods are related to, but still somewhat different from the methods considered in our survey that predict locations of binding sites.*

It would be another way to assess methods by their implementation of true biological mechanisms into their formal approaches. I wonder that biological literature on transcription regulation is not considered in this review. A comprehensive survey is not indicated for this review. But for the purpose of gussing future ways out of the difficulties, one might analyze the experimental data available for a few well-studied transcription complexes and genes regulated by them. Even if a method yet fails to perform in a large-scale test, it might be a good start for further development if its mathematical/analytical formulations captures major mechanistic aspects of the biological process of recognizing regulatory sequences. Another mathematical reformulation of existing approaches will certainly not change the status of the field.

**Author response: ***We completely agree that it would be beneficial to have access to a good state of the art overview over the biological aspects of transcription regulation, from the point of view of motif discovery. However, we feel that such an overview will be outside the scope of this review, and probably more suited as a separate review paper.*

The increasing availability of data from high-throuput methodologies (e.g., microarray (ChIP) data) for certain DNA-binding protein complexes will possibly change the situation for developing prediction tools in the near future.

In its present form, the review can be useful for people in the field since some part of the vast literature is organized in a reasonable way. At the same time, the review does not give guidance to the reader, which lines of prediction tool development are most promising and what conditions must be fulfilled to move the field out of its apparent stagnation.

**Author response: ***Our strong focus on methods using different types of data in an integrated analysis, combined with a critical attention to implementation details, should be read as a guidance to the reader.*

**Figure 1:**
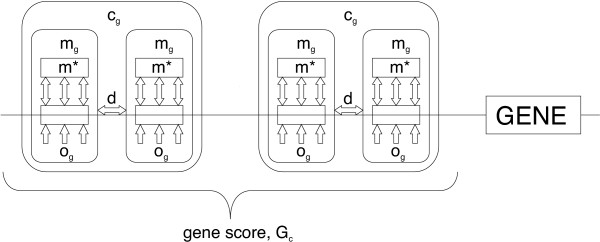
**A schematic view of the integrated framework**. A single motif, denoted by *m*_*g*_, consists of two parts, *m*_*g *_is how well the sequence matches a consensus, while *o*_*g *_is a prior on whether any regulatory element is to occur at that position. A set of single motifs, together with inter-motif distance restrictions (*d*), then forms a composite motif (*c*_*g*_). Finally, multiple occurrences of a composite motif in the regulatory regions of a gene is represented by a gene score *G*_*c*_.

## References

[B1] Korn LJ, Queen CL, Wegman MN (1977). Computer analysis of nucleic acid regulatory sequences. Proc Natl Acad Sci U S A.

[B2] Queen C, Wegman MN, Korn LJ (1982). Improvements to a program for DNA analysis: a procedure to find homologies among many sequences. Nucleic Acids Res.

[B3] Stormo GD, Schneider TD, Gold L, Ehrenfeucht A (1982). of the 'Perceptron' algorithm to distinguish translational initiation sites in E. coli. Nucleic Acids Res.

[B4] Staden R (1984). Computer methods to locate signals in nucleic acid sequences. Nucleic Acids Res.

[B5] Stormo GD (2000). DNA binding sites: representation and discovery. Bioinformatics.

[B6] Pavesi G, Mauri G, Pesole G (2004). In silico representation and discovery of transcription factor binding sites. Brief Bioinform.

[B7] Wasserman WW, Krivan W (2003). In silico identification of metazoan transcriptional regulatory regions. Naturwissenschaften.

[B8] Bulyk ML (2003). Computational prediction of transcription-factor binding site locations. Genome Biol.

[B9] Hannenhalli S, Levy S (2001). Promoter prediction in the human genome. Bioinformatics.

[B10] Tompa M, Li N, Bailey TL, Church GM, De Moor B, Eskin E, Favorov AV, Frith MC, Fu Y, Kent WJ, Makeev VJ, Mironov AA, Noble WS, Pavesi G, Pesole G, Regnier M, Simonis N, Sinha S, Thijs G, van Helden J, Vandenbogaert M, Weng Z, Workman C, Ye C, Zhu Z (2005). Assessing computational tools for the discovery of transcription factor binding sites. Nat Biotechnol.

[B11] van Driel R, Fransz PF, Verschure PJ (2003). The eukaryotic genome: a system regulated at different hierarchical levels. J Cell Sci.

[B12] Werner T (1999). Models for prediction and recognition of eukaryotic promoters. Mamm Genome.

[B13] Wray GA, Hahn MW, Abouheif E, Balhoff JP, Pizer M, Rockman MV, Romano LA (2003). The evolution of transcriptional regulation in eukaryotes. Mol Biol Evol.

[B14] Pedersen AG, Baldi P, Chauvin Y, Brunak S (1999). The biology of eukaryotic promoter prediction–areview. Comput Chem.

[B15] Kato M, Hata N, Banerjee N, Futcher B, Zhang MQ (2004). Identifying combinatorial regulation of transcription factors and binding motifs. Genome Biol.

[B16] Brazma A, Jonassen I, Eidhammer I, Gilbert D (1998). Approaches to the automatic discovery of patterns in biosequences. J Comput Biol.

[B17] Brazma A, Jonassen I, Vilo J, Ukkonen E (1998). Pattern Discovery in Biosequences. ICGI '98:Proceedings of the 4th International Colloquium on Grammatical Inference.

[B18] Table of motif discovery tools. http://www.ntnu.no/~drablos/motif/discovery_tools.html.

[B19] Berg OG, von Hippel PH (1987). Selection of DNA binding sites by regulatory proteins.Statistical-mechanical theory and application to operators and promoters. J Mol Bio l.

[B20] Benos PV, Bulyk ML, Stormo GD (2002). Additivity in protein-DNA interactions: how good an approximation is it?. Nucleic Acids Res.

[B21] Zhou Q, Liu JS (2004). Modeling within-motif dependence for transcription factor binding site predictions. Bioinformatics.

[B22] O'Flanagan RA, Paillard G, Lavery R, Sengupta AM (2005). Non-additivity in protein-DNA binding. Bioinformatics.

[B23] Stormo GD, Schneider TD, Gold L (1986). Quantitative analysis of the relationship between nucleotide sequence and functional activity. Nucleic Acids Res.

[B24] Barash Y, Elidan G, Friedman N, Kaplan T (2003). Modeling dependencies in protein-DNA binding sites. RECOMB '03: Proceedings of the seventh annual international conference on Computational molecular biology.

[B25] Lim LP, Burge CB (2001). A computational analysis of sequence features involved in recognition of short introns. Proc Natl Acad Sci USA.

[B26] Cawley S (2000). Statistical models for DNA sequencing and analysisspliceosome: motors, clocks, springs, and things. Cel 1, Statistical models for DNA sequencing and analysis. PhD thesis.

[B27] Zhao X, Huang H, Speed TP (2004). Finding short DNA motifs using permuted markov models. In. RECOMB '04-' Proceedings of the eighth annual international conference on Computational molecular biology.

[B28] Ben-Gal I, Shani A, Gohr A, Grau J, Arviv S, Shmilovici A, Posch S, Grosse I (2005). Identification of transcription factor binding sites with variable-order Bayesian networks. Bioinformatics.

[B29] Xing EP, Jordan MI, Karp RM, Russell S, Becker S, Thrun S, Obermayer K (2002). A hierarchical bayesian markovian model for motifs in biopolymer sequences. Advances in Neural Information Processing Systems.

[B30] Kechris KJ, van Zwet E, Bickel PJ, Eisen MB (2004). Detecting DNA regulatory motifs by incorporating positional trends in information content. Genome Biol.

[B31] van Helden J, Andre B, Collado-Vides J (1998). Extracting regulatory sites from the upstream region of yeast genes by computational analysis of oligonucleotide frequencies. J Mol Biol.

[B32] Jensen LJ, Knudsen S (2000). Automatic discovery of regulatory patterns in promoter regions based on whole cell expression data and functional annotation. Bioinformatics.

[B33] Sinha S, Tompa M (2000). A statistical method for finding transcription factor binding sites. Proc Int Conf Intell Syst Mol Biol.

[B34] Bussemaker HJ, Li H, Siggia ED (2000). Building a dictionary for genomes: identification of presumptive regulatory sites by statistical analysis. Proc Natl Acad Sci USA.

[B35] van Helden J, Rios AF, Collado-Vides J (2000). Discovering regulatory elements in non-coding sequences by analysis of spaced dyads. Nucleic Acids Res.

[B36] Shinozaki D, Maruyama O (2002). A Method for the Best Model Selection for Single and Paired Motifs. Genome Informatics.

[B37] Takusagawa KT, Gifford DK (2004). Negative information for motif discovery. Pac Symp Biocomput.

[B38] Xie X, Lu J, Kulbokas EJ, Golub TR, Mootha V, Lindblad-Toh K, Lander ES, Kellis M (2005). Systematic discovery of regulatory motifs in human promoters and 3' UTRs by comparison of several mammals. Nature.

[B39] Tompa M (1999). An exact method for finding short motifs in sequences, with application to the ribosome binding site problem. Proc Int Conf Intell Syst Mol Biol.

[B40] Marsan L, Sagot MF (2000). Algorithms for extracting structured motifs using a suffix tree with an application to promoter and regulatory site consensus identification. J Comput Biol.

[B41] Pevzner PA, Sze SH (2000). Combinatorial approaches to finding subtle signals in DNA sequences. Proc Int Conf Intell Syst Mol Biol.

[B42] Pavesi G, Mauri G, Pesole G (2001). An algorithm for finding signals of unknown length in DNA sequences. Bioinformatics.

[B43] Eskin E, Pevzner PA (2002). Finding composite regulatory patterns in DNA sequences. Bioinformatics.

[B44] Baldwin NE, Collins RL, Langston MA, Leuze MR, Symons CT, Voy BH (2004). High performance computational tools for motif discovery. 18th International Parallel and Distributed Processing Symposium (IPDPS'04) – Workshop 9.

[B45] Li HL, Fu CJ (2005). A linear programming approach for identifying a consensus sequence on DNA sequences. Bioinformatics.

[B46] Blanchette M, Tompa M (2002). Discovery of regulatory elements by a computational method for phylogenetic footprinting. Genome Res.

[B47] Jensen ST, Liu XS, Liu JS, Zhou Q (2004). Computational Discovery of Gene Regulatory Binding Motifs: A Bayesian Perspective. Statist Sci.

[B48] Sinha S, van Nimwegen E, Siggia ED (2003). A probabilistic method to detect regulatory modules. Bioinformatics.

[B49] Liu X, Brutlag DL, Liu JS (2001). BioProspector: discovering conserved DNA motifs in upstream regulatory regions of co-expressed genes. Pac Symp Biocomput.

[B50] Donaldson IJ, Chapman M, Gottgens B (2005). TFBScluster: a resource for the characterisation of transcriptional regulatory networks. Bioinformatics.

[B51] McCue L, Thompson W, Carmack C, Ryan MP, Liu JS, Derbyshire V, Lawrence CE (2001). Phylogenetic footprinting of transcription factor binding sites in proteobacterial genomes. Nucleic Acids Res.

[B52] Thompson W, Rouchka EC, Lawrence CE (2003). Gibbs Recursive Sampler: finding transcription factor binding sites. Nucleic Acids Res.

[B53] Tagle DA, Koop BF, Goodman M, Slightom JL, Hess DL, Jones RT (1988). Embryonic epsilon and gamma globin genes of a prosimian primate (Galago crassicaudatus). Nucleotide and amino acid sequences, developmental regulation and phylogenetic footprints. J Mol Biol.

[B54] Zhang Z, Gerstein M (2003). Of mice and men: phylogenetic footprinting aids the discovery of regulatory elements. J Biol.

[B55] Krivan W, Wasserman WW (2001). A predictive model for regulatory sequences directing liver-specific transcription. Genome Res.

[B56] Sharan R, Ovcharenko I, Ben-Hur A, Karp RM (2003). CREME: a framework for identifying cis-regulatory modules in human-mouse conserved segments. Bioinformatics.

[B57] Cora D, Herrmann C, Dieterich C, Di Cunto F, Provero P, Caselle M (2005). Ab initio identification of putative human transcription factor binding sites by comparative genomics. BMC Bioinformatics.

[B58] Wasserman WW, Fickett JW (1998). Identification of regulatory regions which confer muscle-specific gene expression. J Mol Biol.

[B59] Wang T, Stormo GD (2005). Identifying the conserved network of cis-regulatory sites of a eukaryotic genome. Proc Natl Acad Sci USA.

[B60] Beiko RG, Charlebois RL (2005). GANN: genetic algorithm neural networks for the detection of conserved combinations of features in DNA. BMC Bioinformatics.

[B61] Pudimat R, Schukat-Talamazzini EG, Backofen R (2004). Feature Based Representation and Detection of Transcription Factor Binding Sites. Proceedings of the German Conference on Bioinformatics.

[B62] Ponomarenko JV, Ponomarenko MP, Frolov AS, Vorobyev DG, Overton GC, Kolchanov NA (1999). Conformational and physicochemical DNA features specific for transcription factor binding sites. Bioinformatics.

[B63] El Hassan MA, Calladine CR (1997). Conformational characteristics of DNA: empirical classifications and a hypothesis for the conformational behaviour of dinucleotide steps. Roy Soc of London Phil Tr A.

[B64] Kel A, Kel-Margoulis O, Ivanova T, Wingender E (2001). ClusterScan: A Tool for Automatic Annotation of Genomic Regulatory Sequences by Searching for Composite Clusters. Proceedings of the German Conference on Bioinformatics.

[B65] Hu YJ (2003). Finding subtle motifs with variable gaps in unaligned DNA sequences. Comput Methods Programs Biomed.

[B66] Thompson W, Palumbo MJ, Wasserman WW, Liu JS, Lawrence CE (2004). Decoding human regulatory circuits. Genome Res.

[B67] Bussemaker HJ, Li H, Siggia ED (2001). Regulatory element detection using correlation with expression. Nat Genet.

[B68] GimaThakurta D, Stormo GD (2001). Identifying target sites for cooperatively binding factors. Bioinformatics.

[B69] Rebeiz M, Reeves NL, Posakony JW (2002). SCORE: a computational approach to the identification of cis-regulatory modules and target genes in whole-genome sequence data. Site clustering over random expectation. Proc Natl Acad Sci USA.

[B70] Aerts S, Van Loo P, Thijs G, Moreau Y, De Moor B (2003). Computational detection of cis -regulatory modules. Bioinformatics.

[B71] Bailey TL, Noble WS (2003). Searching for statistically significant regulatory modules. Bioinformatics.

[B72] Frith MC, Hansen U, Weng Z (2001). Detection of cis-element clusters in higher eukaryotic DNA. Bioinformatics.

[B73] Xing EP, Wu W, Jordan MI, Karp RM (2004). Logos: a modular bayesian model for de novo motif detection. J Bioinform Comput Biol.

[B74] Gupta M, Liu JS (2005). De novo cis-regulatory module elicitation for eukaryotic genomes. Proc Natl Acad Sci USA.

[B75] Wagner A (1999). Genes regulated cooperatively by one or more transcription factors and their identification in whole eukaryotic genomes. Bioinformatics.

[B76] Frech K, Werner T (1997). Specific modelling of regulatory units in DNA sequences. Pac Symp Biocomput.

[B77] Scherf M, Klingenhoff A, Werner T (2000). Highly specific localization of promoter regions in large genomic sequences by Promoterlnspector: a novel context analysis approach. J Mol Biol.

[B78] Brazma A, Vilo J, Ukkonen E, Valtonen K (1997). Data mining for regulatory elements in yeast genome. Proc Int Conf Intell Syst Mol Biol.

[B79] Policriti A, Vitacolonna N, Morgante M, Zuccolo A (2004). Structured motifs search. RECOMB '04: Proceedings of the eighth annual international conference on Computational molecular biology.

[B80] Berman BP, Nibu Y, Pfeiffer BD, Tomancak P, Celniker SE, Levine M, Rubin GM, Eisen MB (2002). Exploiting transcription factor binding site clustering to identify cis-regulatory modules involved in pattern formation in the Drosophila genome. Proc Natl Acad Sci USA.

[B81] Segal E, Barash Y, Simon I, Friedman N, Koller D (2002). From promoter sequence to expression: a probabilistic framework. RECOMB '02: Proceedings of the sixth annual international conference on Computational biology.

[B82] Aerts S, Van Loo P, Moreau Y, De Moor B (2004). A genetic algorithm for the detection of new cis-regulatory modules in sets of coregulated genes. Bioinformatics.

[B83] Klingenhoff A, Freeh K, Quandt K, Werner T (1999). Functional promoter modules can be detected by formal models independent of overall nucleotide sequence similarity. Bioinformatics.

[B84] Johansson O, Alkema W, Wasserman WW, Lagergren J (2003). Identification of functional clusters of transcription factor binding motifs in genome sequences: the MSCAN algorithm. Bioinformatics.

[B85] Mahony S, Hendrix D, Golden A, Smith TJ, Rokhsar DS (2005). Transcription factor binding site identification using the self-organizing map. Bioinformatics.

[B86] Workman CT, Stormo GD (2000). a method for discovering transcription factor binding sites with improved specificity. Pac Symp Biocomput.

[B87] Liu XS, Brutlag DL, Liu JS (2002). An algorithm for finding protein-DNA binding sites with applications to chromatin-immunoprecipitation microarray experiments. Nat Biotechnol.

[B88] Wang T, Stormo GD (2003). Combining phylogenetic data with co-regulated genes to identify regulatory motifs. Bioinformatics.

[B89] Conlon EM, Liu XS, Lieb JD, Liu JS (2003). Integrating regulatory motif discovery and genome-wide expression analysis. Proc Natl Acad Sci USA.

[B90] Frith MC, Fu Y, Yu L, Chen JF, Hansen U, Weng Z (2004). Detection of functional DNA motifs via statistical over-representation. Nucleic Acids Res.

[B91] Caselle M, Di Cunto F, Provero P (2002). Correlating overrepresented upstream motifs to gene expression: a computational approach to regulatory element discovery in eukaryotes. BMC Bioinformatics.

[B92] Cora D, Di Cunto F, Provero P, Silengo L, Caselle M (2004). Computational identification of transcription factor binding sites by functional analysis of sets of genes sharing overrepresented upstream motifs. BMC Bioinformatics.

[B93] Curran MD, Liu H, Long F, Ge N (2003). Statistical methods for joint data mining of gene expression and DNA sequence database. SIGKDD Explor Newsl.

[B94] Segal E, Yelensky R, Koller D (2003). Genome-wide discovery of transcriptional modules from DNA sequence and gene expression. Bioinformatics.

[B95] Hong P, Liu X, Zhou Q, Lu X, Liu JS, Wong WH (2005). A boosting approach for motif modeling using ChlP-chip data. Bioinformatics.

[B96] Gupta M, Liu JS (2003). Discovery of Conserved Sequence Patterns Using a Stochastic Dictionary Model. Journal of the American Statistical Association.

[B97] Lawrence CE, Altschul SF, Boguski MS, Liu JS, Neuwald AF, Wootton JC (1993). Detecting subtle sequence signals: a Gibbs sampling strategy for multiple alignment. Science.

[B98] Bailey TL, Elkan C (1995). The value of prior knowledge in discovering motifs with. Proc Int Conf Intell Syst Mol Biol.

[B99] Jonassen I (1997). Efficient discovery of conserved patterns using a pattern graph. Comput ApplBiosci.

[B100] Rigoutsos I, Floratos A (1998). Combinatorial pattern discovery in biological sequences: The TEIRESIAS algorithm. Bioinformatics.

[B101] Rustici G, Mata J, Kivinen K, Lio P, Penkett CJ, Burns G, Hayles J, Brazma A, Nurse P, Bahler J (2004). Periodic gene expression program of the fission yeast cell cycle. Nat Genet.

[B102] Birnbaum K, Benfey PN, Shasha DE (2001). cis element/transcription factor analysis (cis/TF): a method for discovering transcription factor/cis element relationships. Genome Res.

[B103] Zhu Z, Pilpel Y, Church GM (2002). Computational identification of transcription factor binding sites via a transcription-factor-centric clustering (TFCC) algorithm. J Mol Biol.

[B104] Ren B, Robert F, Wyrick JJ, Aparicio O, Jennings EG, Simon I, Zeitlinger J, Schreiber J, Hannett N, Kanin E, Volkert TL, Wilson CJ, Bell SP, Young RA (2000). Genome-wide location and function of DNA binding proteins. Science.

[B105] Buck MJ, Lieb JD (2004). ChlP-chip: considerations for the design, analysis, and application of genome-wide chromatin immunoprecipitation experiments. Genomics.

[B106] Mironov AA, Koonin EV, Roytberg MA, Gelfand MS (1999). Computer analysis of transcription regulatory patterns in completely sequenced bacterial genomes. Nucleic Acids Res.

[B107] Qin ZS, McCue LA, Thompson W, Mayerhofer L, Lawrence CE, Liu JS (2003). Identification of co-regulated genes through Bayesian clustering of predicted regulatory binding sites. Nat Biotechnol.

[B108] McGuire AM, Hughes JD, Church GM (2000). Conservation of DNA regulatory motifs and discovery of new motifs in microbial genomes. Genome Res.

[B109] Thijs G, Marchal K, Lescot M, Rombauts S, De Moor B, Rouze P, Moreau Y (2002). A Gibbs sampling method to detect overrepresented motifs in the upstream regions of coexpressed genes. J Comput Biol.

[B110] Park PJ, Butte AJ, Kohane IS (2002). Comparing expression profiles of genes with similar promoter regions. Bioinformatics.

[B111] Holmes I, Bruno WJ (2000). Finding regulatory elements using joint likelihoods for sequence and expression profile data. Proc Int Conf Intell Syst Mol Biol.

[B112] Bar-Joseph Z, Gerber GK, Lee TI, Rinaldi NJ, Yoo JY, Robert F, Gordon DB, Fraenkel E, Jaakkola TS, Young RA, Gifford DK (2003). Computational discovery of gene modules and regulatory networks. Nat Biotechnol.

[B113] Evans PA, Smith AD (2003). Toward optimal motif enumeration. Proceedings of Workshop on Algorithms and Data Structures (WADS 2003).

[B114] Lawrence CE, Reilly AA (1990). An expectation maximization (EM) algorithm for the identification and characterization of common sites in unaligned biopolymer sequences. Proteins.

[B115] Sinha S, Blanchette M, Tompa M (2004). PhyME: a probabilistic algorithm for finding motifs in sets of orthologous sequences. BMC Bioinformatics.

[B116] Prakash A, Blanchette M, Sinha S, Tompa M (2004). Motif discovery in heterogeneous sequence data. Pac Symp Biocomput.

[B117] Ao W, Gaudet J, Kent WJ, Muttumu S, Mango SE (2004). Environmentally induced foregut remodeling by PHA-4/FoxA and DAF-12/NHR. Science.

[B118] Neuwald AF, Liu JS, Lawrence CE (1995). Gibbs motif sampling: detection of bacterial outer membrane protein repeats. Protein Sci.

[B119] Zhou Q, Wong WH (2004). CisModule: de novo discovery of cis-regulatory modules by hierarchical mixture modeling. Proc Natl Acad Sci USA.

[B120] Grad YH, Roth FP, Halfon MS, Church GM (2004). Prediction of similarly-acting cis-regulatory modules by subsequence profiling and comparative genomics in D. melanogaster and D. pseudoobscura. Bioinformatics.

[B121] Hart RK, Royyuru AK, Stolovitzky G, Califano A (2000). Systematic and fully automated identification of protein sequence patterns. J Comput Biol.

[B122] Favorov AV, Gelfand MS, Gerasimova AV, Ravcheev DA, Mironov AA, Makeev VJ (2005). A Gibbs sampler for identification of symmetrically structured, spaced DNA motifs with improved estimation of the signal length. Bioinformatics.

[B123] Marsan L, Sagot MF (2000). Extracting structured motifs using a suffix treealgorithms and application to promoter consensus identification. RECOMB '00: Proceedings of the fourth annual international conference on Computational molecular biology.

[B124] Roth FP, Hughes JD, Estep PW, Church GM (1998). Finding DNA regulatory motifs within unaligned noncoding sequences clustered by whole-genome mRNA quantitation. Nat Biotechnol.

